# Neuroendocrine neoplasms of gastrointestinal tract and secondary primary synchronous tumors: A systematic review of case reports. Casualty or causality?

**DOI:** 10.1371/journal.pone.0216647

**Published:** 2019-05-14

**Authors:** Rafael Parra-Medina, Paula Moreno-Lucero, Julian Jimenez-Moreno, Alejandra María Parra-Morales, Alfredo Romero-Rojas

**Affiliations:** 1 Research Institute, Fundación Universitaria de Ciencias de la salud, Bogotá, Colombia; 2 Department of Pathology, Fundación Universitaria de Ciencias de la salud, Bogotá, Colombia; 3 Department of Pathology, National Institute of Cancer, Bogotá, Colombia; Istituto di Ricovero e Cura a Carattere Scientifico Centro di Riferimento Oncologico della Basilicata, ITALY

## Abstract

**Background:**

Neuroendocrine neoplasms (NENs) can arise in most of the epithelial organs of the body and are not a rare condition in the gastrointestinal tract (GIT). The presence of NENs in GIT associated with other secondary primary malignancies (SPM) has been considered an exotic event. This study aims to describe the case reports of NENs accompanied by synchronous primary tumors.

**Methods and findings:**

We performed a systematic literature search of the databases Scopus, PubMed, Scielo and LILACS to identify case reports that described the presence of NENs in GIT with SPM. 78 case reports were included. The mean of age of the cases was 60.2 years. 60% were male. 95.4% were NENs G1. 17 cases of NENs had metastasis. 80% of SPM were recognized in the GIT (36% in stomach, 27% in large intestine, 11.2% in small intestine, and 5.6% in esophagus). The most common type of SPM was adenocarcinoma (49.4%), followed by GIST (13.5%), other NENs in different GIT segment (7.9%), lymphoma (6.8%), and squamous cell carcinoma (4.5%). The most common tumor in GIT was adenocarcinoma (97.6%) and the presence of adenocarcinoma in the same segment of GIT was found in 68.4% of the cases. Association between adenocarcinomas and NENs in GIT (p:<0.0001) and adenocarcinoma and tumor in the same segment of GIT location were observed (p<0.001).

**Conclusion:**

These results demonstrate that NENs with SPM are not a rare condition. Several theories have been proposed to explain this association; one of these is the ability of NENs to generate synchronous tumors by autocrine and paracrine effect. We observed an association between adenocarcinomas and NENs in the same segment of GIT.

## Introduction

Neuroendocrine neoplasms (NENs) can arise in most of the epithelial organs of the body. NENs are a diverse group of tumors and have been found predominantly in the lung, tubular gastrointestinal tract, and pancreas. The terminology for NENs has been problematic for various reasons [1–3]. The current classification (2018) aims to stratify NENs by their prognosis. This classification adapted by World Health Organization (WHO) is divided according to the anatomic site (tubular gastrointestinal tract (GIT), pancreas, uterus, and lung); family (Neuroendocrine tumors (NETs) or Neuroendocrine carcinoma (NEC)); and grade. In GIT the current terminology of NETs are grade 1 (G1), grade 2 (G2), and grade 3 (G3), while the NECs are classified as small cell neuroendocrine carcinoma and large cell neuroendocrine carcinoma [3].

NENs account for about of 0.5% of newly diagnosed neoplasms [4]. Their incidence has increased, possibly due to improved diagnostic techniques [5]. Their prevalence in the United States is estimated at 103.312 cases, which is twice the prevalence of pancreatic and gastric cancers combined [5,6]. These tumors have a female preponderance of around 2.5:1, and the most frequent primary sites are the gastrointestinal tract (62%-67%) and the lung (22%-27%). 12% to 22% of patients are metastatic at presentation [4]. The most commonly documented NENs locations in GIT are colon and rectum (69%), followed by small intestine (36%), stomach (10%), appendix (5%) and esophagus (0.4–2%) [7,8]

The majority of NENs arise sporadically, but an association with Multiple Endocrine Neoplasia Syndrome type 1 and familial clustering is recognized [4]. NENs can be found synchronous (occurring at the same time) with other secondary primary malignancies (SPM) or metachronous (occurring at different times) [9–12], even without genetic predisposition syndrome [12]. The case reports of these occurrences have increased over time and have been considered as unusual. Therefore, the aim of this systematic review is to describe the cases of NENs accompanied with synchronous tumors reported in the literature.

## Methods

### Search strategy

We carried out a systematic review of case reports published in Scopus, PubMed, Scielo and LILACS, which includes BIREME, and many other LA sources. No limits regarding language or publication period were placed on the search. The search was done using the following terms: *synchronous AND (neuroendocrine tumors OR carcinoid tumors OR small cell carcinoma OR large cell carcinoma)*. The PRISMA guidelines were followed during data extraction, analysis, and reporting [13]. The search was done during June 2018. We considered papers available in the following languages: English and Spanish.

### Study selection, data extraction and quality assessment

The inclusion criteria for the systematic review were the following: case reports with the presence of NENs confirmed by histopathology in the GIT with the presence of synchronous malignant tumors in any location. SPM were defined as any primary malignant tumor which occurred at the same time with the other tumor. The exclusion criteria were the presence of mixed adenoneuroendocrine carcinomas (MANECs) or neuroendocrine carcinoma of unknown primary site.

Two reviewers screened all the titles and abstracts from the publications performed an eligibility assessment. Retrieved articles were rejected if the eligibility criteria were not met. A third reviewer was consulted when eligibility criteria were unclear. Hand-searches were performed from the articles that seemed to be relevant.

The extracted data from each article were: author name, year of publication, age, gender, localization of NENs (esophagus, stomach, small intestine (duodenum, jejunum and ileum) and colorectum (appendix, colon and rectum), family of NENs, grade of NENs and localization and type of SPM, and presence of metastasis of NENs and SPM.

To assess the article quality we used a modified tool version that has been used in previous studies [14,15]. Three researchers independently evaluated the assessments of all case reports. We used three items: (1) Patients were described adequately (clinical history, laboratory and radiological findings); (2) accurate diagnoses were provided (Diagnosis of NENs and SPM were confirmed by pathology); (3) convincing evidence in support of the diagnosis was presented (NENs and SPM were confirmed using immunohistochemistry or the pathological images showed in the article, the images were interpreted by a pathologist (RPM)). Data extracted by the abstract were not included in the quality assessment.

### Data synthesis and analysis

Univariate analysis was applied to determine distribution of clinical and pathological findings. Type and location of SPM were analyzed as a different case report in patients with more than one SPM. Patients with multiple NENs tumors in the same localization were analyzed as one only case report; this was done in order to avoid overestimation. Chi-square test was employed to determine statistically significant differences between localization of NENs and tumor subtype. A p-value <0.05 was considered statistically significant. Statistical analysis was performed with the STATA 13.

## Results

### Systematic review literature

**Fig 1:**
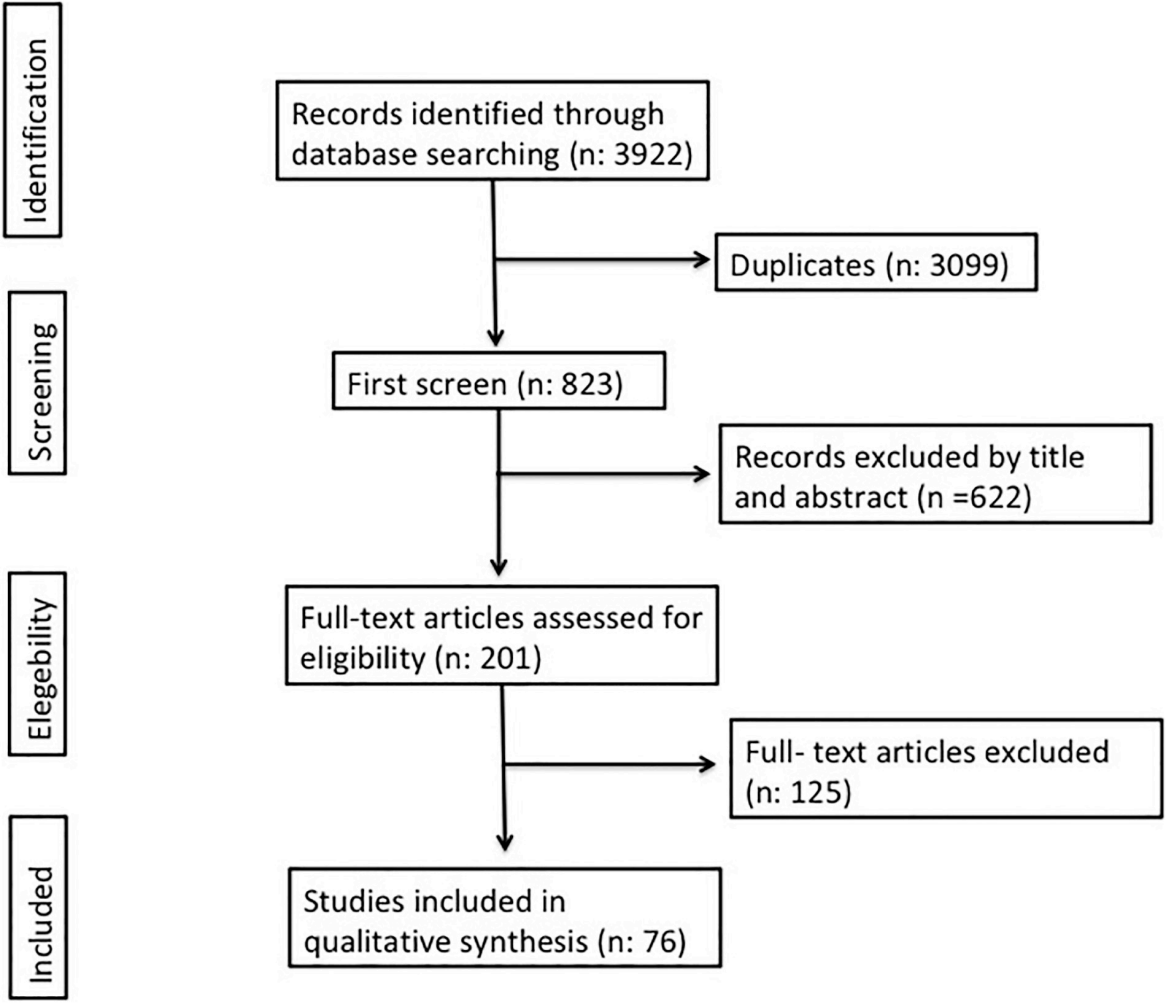
Flow chart of the systemic literature review.

We identified 3922 articles in the databases. Of these, 3099 were identified as duplicates. A total of 201 full text articles were assessed for eligibility. Finally, 76 articles that contained interpretable data and fulfilled the eligibility criteria were included (Fig 1). [16–90] In 16 articles the data extraction was made from its abstract [21,22,24,33–35,37,45,64,70,71,73,74,80]. The flowchart for systematic literature review and articles included in the analysis is shown in Fig 1. Detailed information is shown in the S1 Table.

### Quality appraisal

The overall quality of the cases was good. Most cases reported an adequate description of patient past medical history, laboratory and radiology findings (98%). Accurate diagnosis was provided in 95% and convincing evidence of diagnosis was provided in 70% (S2 Table).

### Patient characteristics

The mean of age of the cases was 60.2 years old (standard deviation 14 years). 60% were male. 77 cases reports had NENs of the GIT (S1 Table). Three in the esophagus, 16 in the stomach, 36 in the small intestine, 22 in the large intestine (16 in the colon and rectum and 6 in the appendix). 56 cases were NET G1, two cases were NET G2, 3 cases were NET G3, and in one case the grade of NET was not available. 15 cases were NEC (10 small cell neuroendocrine carcinoma and 5 large cell neuroendocrine carcinoma). 17 cases of NENs had metastasis (11 cases of NETs and 6 NEC) (Table 1). And six cases had metastasis of the SPM (4 adenocarcinomas, 1 glioblastoma and 1 NET G1). Eight cases had more than one SPM [18,32,34,35,53,57,68,70] (these cases were added to the statistical analysis as a single case report) (S3 Table). And six cases had more than one NENs in the same GIT (these cases were analysis as one only case report) [26,37,41,49,51,61]. In total 89 case reports were included in the statistical analysis.

**Table 1:** Characteristic of patients with NENs.

	NET G1	NET G2	NET G3	NET grade non available	NEC (small cell)	NEC (large cell)	Total
Total	56	2	3	1	10	5	77
Age (mean)	60.2 years	62.5 years	74 years	52 years	48.6 years	62.4 years	60.2 years
*Gender*							
Male	31	1	2	1	6	5	46
Female	21	1	1	0	4	0	27
Non available	4	0	0	0	0	0	4
*Location*							
Esophagus	0	0	0	0	3	0	3
Stomach	13	0	0	0	0	3	16
Small intestine	30	2	2	0	2	2	36
Appendix	6	0	0	0	0	0	6
Large intestine	7	0	1	1	5	0	16
*Metastasis*							
Yes	8	1	1	1	3	3	17
No	43	1	2	0	6	1	53
Non available	5	0	0	0	1	1	7

From the rare cases with atypical location and patients with more than one SPM (S3 Table), we also found cases with malignant tumors and benign tumors [59,86]. Furthermore, we also found cases with other conditions such as microsatellite instability [50,63], intestinal inflammatory disease [42,50,80] and neurofibromatosis [53,60].

### Location and type of tumors in the SPM

We found that 80% of SPM were recognized in the GIT (36% in the stomach, 27% in the large intestine, 11.2% in the small intestine, and 5.6% in the esophagus). The other SPM were present in different sites (4.5% in the lung, 3.4% in the kidney, 2.2% in brain and pancreas, and 1.1% case in ovary, lymph node, cervix, bone marrow, ureter, liver, and prostate) (Table 2 and S1 Table).

**Table 2:** Location and type of tumor of SPM.

Location	Adenocarcinoma	GIST	NENs	SCC	Lymphoma	Unclassifiable Carcinoma	RCC	Glioblastoma	Non small cell lung cancer	Rhabdomyosarcoma	Steroid cell tumor	Angiosarcoma	HCC	Heary cell leukemia	Total
Esophagous	1			3		1									5
Stomach	17	10	2	1	2										32
Small intestine	3	2	2		3										10
Large intestine	21		1			2									24
Bone marrow	0													1	1
Brain	0							2							2
Cervix	0									1					1
Lung	1		1						1			1			4
Lymph node	0				1										1
Ovary	0										1				1
Pancreas	0		1			1									2
Liver	0												1		1
Kidney	0						3								3
Prostate	1														1
Ureter						1									1
Total	44	12	7	4	6	5	3	2	1	1	1	1	1	1	89

Abbreviations: HCC: Hepatocellular carcinoma; GIST: Gastrointestinal Stromal Tumor; RCC: Renal cell carcinoma; SCC: Squamous cell carcinoma.

The most common type of SPM were adenocarcinomas (49.4%), followed by GIST (13.5%), other NENs in different GIT segments (7.9%), lymphomas (6.8%), squamous cell carcinoma (4.5%), unclassifiable carcinomas (5.6%), renal cell carcinomas (3.4%), glioblastomas (2.2%), angiosarcoma (1.1%), non-small cell lung (1.1%), rhabdomyosarcoma (1.1%), ovarian steroid cell tumor (1.1%) and heary cell leukemia (1.1%), and hepatocellular carcinoma (1.1%) (Table 2 and S1 Table).

The adenocarcinomas were detected in the 95.4% of tumors of the GIT (the colon (47.7%), the stomach (38.6%), the small intestine (6.8%), the esophagus (2.3%)) and some cases in the lung (n: 2.3%), and the prostate (2.3% (Table 3)). While, the GISTs were present in the stomach (83%) and the small intestine (17%), other NENs were observed in the stomach (28%), the small intestine (28%), the colon (14%), the lung (14%) and the pancreas (14%) (Table 4). Lymphomas were present in the small intestine (50%) (MALT (n:1), Follicular lymphoma (n:1) and T-cell lymphoma (n:1)), stomach (33%) (MALT (n:2), and lymph node (17%) (Lymphoplasmacytic lymphoma). Squamous cell carcinomas were present in the esophagus (75%) and the stomach (25%) (Table 2 and S1 Table). On the other hand, the presence of NENs and SPM in the same GIT segment was observed in 43.2% of the cases. 68.4% of the adenocarcinomas were present in the same GIT segment.

**Table 3:** Characteristics of the adenocarcinomas cases.

NENs location	Author	NENs grade	Location of the adenocarcinomas cases				
			Esophagus	Stomach	Small intestine	Large intestine	Lung
Esophagus	Saw E.	NEC (Small cell NE carcinoma)	1				
Stomach	Rassidakis GZ.	NETs G1					
	Cunha P.	NETs G1		1			
	Kim EY.	NETs G1		1			
	Olinici CD.	NETs G1		1			
	Yang L.	NETs G1		1			
	Yasuda K.	NETs G1		1			
	Sawalakhe NR.	NETs G1		1			
	Moya Valverde E.	NETs G1		1			
	Nakayama Y.	NETs G1		1			
	Herreros-Villanueva M.	NEC (Large cell NE carcinoma)		1			
	Muto M	NEC (Large cell NE carcinoma)		1			
Small intestine	Ott RA.	NETs G1				1	
	Rivadeneira D.	NETs G1				1	
	Tse V.	NETs G1				1	
	McCabe H.	NETs G1		1			
	Cioffi U.	NETs G1			1		
	Srilatha PS.	NETs G1		1			
	Reim D.	NETs G1					1
	Aslam M.	NETs G1				1	
	McHugh S.	NETs G1				1	
	Boltin D.	NETs G1			1		
	Wohadlo Ł.	NETs G1				1	
	Martínez MM.	NETs G1		1			
	Cokmert S.	NETs G3				1	
	Fukaya M	NEC (Small cell NE carcinoma)		1			
	Waldon K	NETs G1				1	
	Almajano EA.	NETs G1					
	Kim SH.	NETs G1		1		2	
Large intestine	Park JS.	NEC (Large cell NE carcinoma)				1	
	Gemeinhardt M.	NEC (Small cell NE carcinoma)		1			
	Nakayama Y.	Non available				1	
	Lipka S.	NEC (Small cell NE carcinoma)				1	
	Zhu JG.	NETs G1				1	
	Meeks MW.	NETs G1				1	
	Mohapatra M.	NETs G1				1	
	Vootla V.	NETs G1				1	
	Zukanović G.	NEC (Small cell NE carcinoma)				1	
	Winn JN.	NETs G1				1	
	Winn JN.	NETs G1				1	
Total			1	17	3	21	1

**Table 4:** Characteristics of the GISTs cases.

NENs location	Author	NENs grade	Location of the GISTs cases			
			Esophagus	Stomach	Small intestine	Large intestine
Stomach	Ott Ra	NETs G1		1		
	Cirillo F.	NETs G1		1		
	Samaras VD.	NETs G1		1		
	Duman DG.	NETs G1		1		
	Ding J.	NEC (Large cell NE carcinoma)		1		
Small intestine	Karatzas G.	NETs G1			1	
	Buragas M.	NETs G1		1		
	Koçer NE.	NETs G1			1 *	
	Pusiol T.	NETs G1		1		
	Kaur R.	NETs G1		1		
Total			0	8	2	0

* GIST rich in osteoclast-like giant cells.

The univariate analysis confirmed association between adenocarcinomas and NENs in the GIT (p:<0.0001), and adenocarcinoma and tumor in the same segment of GIT location (p:0.001).

## Discussion

The association of NENs with synchronic tumors is not a rare condition. In 1994 Pearson and Fitzgerald reported, for the first time, a high incidence of carcinoid tumors with SPM in an autopsy series [91]. Large series of cases have documented the presence of NETs G1 (carcinoids) in GIT with synchronous SPM in a range between 62–88% and metachronous tumor between 12–38% [11]. Godwin [92], observed 62% with ≥ 1 synchronic tumors in 2471 patients, while Saha et al [93] found in 67% the secondary malignancy in 112 patients with gastrointestinal NETs G1 (carcinoids). Of such lesions, 53% were adenocarcinoma of the GIT (39% in colon and rectum; 7% in small bowel and 7% in stomach); the remainder occurred in the lung (7%), prostate (7%), cervix (7%), and other diverse sites. We found that NETs G1 associated with SPM in GIT were present in 54% (S1 Table). 80% of SPM were recognized in the GIT (45% in stomach, 34% in large intestine, 14% in small intestine and 7% in esophagus). The remainder occurred in other sites (lung (4.5%), kidney (3.4%) brain (2.2%), pancreas (2.2%), ovary (1.1%), lymph node (1.1%), cervix (1.1%), bone marrow (1.1%), ureter (1.1%), liver (1.1%), and prostate (1.1%)). In addition, the most frequent SPM was adenocarcinoma (colon (48%), stomach (38%), small intestine (7%), esophagus (2.2%), lung (2.2%), and prostate (2.2%)).

The most common NENs in the GIT are located in the colon and rectum (69%), followed by small intestine (36%), stomach (10%), appendix (5%) and, less frequently, in the esophagus (0.4–2%) [7,8] We found most cases in the small intestine (46%), followed by the colon and rectum (21.8%), the stomach (20.5%), appendix (7.7%), and the esophagus (3.8%) (Table 1). All of NECs of esophagus were small cell neuroendocrine carcinoma and co-existence with adenocarcinoma or SCC in the esophagus [8,94] Huang et al. reported that over 80% of NEC of the esophagus has synchronous SCC including tumor in situ [94]. We found two cases of small cell neuroendocrine carcinoma with SCC synchronic in esophagus: one case with adenocarcinoma [16] and the other case GIST and adenocarcinoma in stomach [18].

Very rare cases of SPM were observed in the present study. For example the presence of poorly differentiated SCC in stomach [83] (the annual incidence rate is 0.04–0.07% [95]); We observed the following: a case with angiosarcoma of the lung, GIST in the stomach, adenocarcinoma in the prostate and rectum, and NET G1 in the small intestine [35]; a case of a 59-year-old woman with NET G2 of the terminal ileum with metastasis to a mesenteric lymph node and a steroid cell tumor of the left ovary [65]; a case of 43-year-old woman with embryonal rhabdomyosarcoma of the cervix and incidental NET G1 in the appendix [78]. Also, we found a rare histological subtype in one case of a GIST with osteoclast-like giant cells and NET G1 in Ampulla of Vater [43].

Six cases of GIT lymphoma were observed (four MALT, one Follicular lymphoma and one T-cell lymphoma). And in three cases the lymphomas were present in the same intestinal segment where NEN was. We found a case with Follicular lymphoma and NET G1 in the Ampulla of Vater [52]. Two cases had MALT, one case in small intestine with NET G1 [58], and other case in stomach with NEC (Large cell NE carcinoma) and adenocarcinoma [32]. The presence of synchronic adenocarcinoma and lymphoma in the GIT is a rare condition [96,97].

The pathogenesis of NENs associated with SPM remains unclear. Diverse theories have been developed like the existence of a common carcinogenic effect that stimulates the growth of NETS and secondary primary tumor [11] or a common stem cell which may undergo similar genetic mutations (e.g. c-kit or p53) and give rise to different types of gastrointestinal malignancies [8,98,99]. Curiously, positivity of NENs markers has been observed in other carcinomas [100–102].

**Fig 2:**
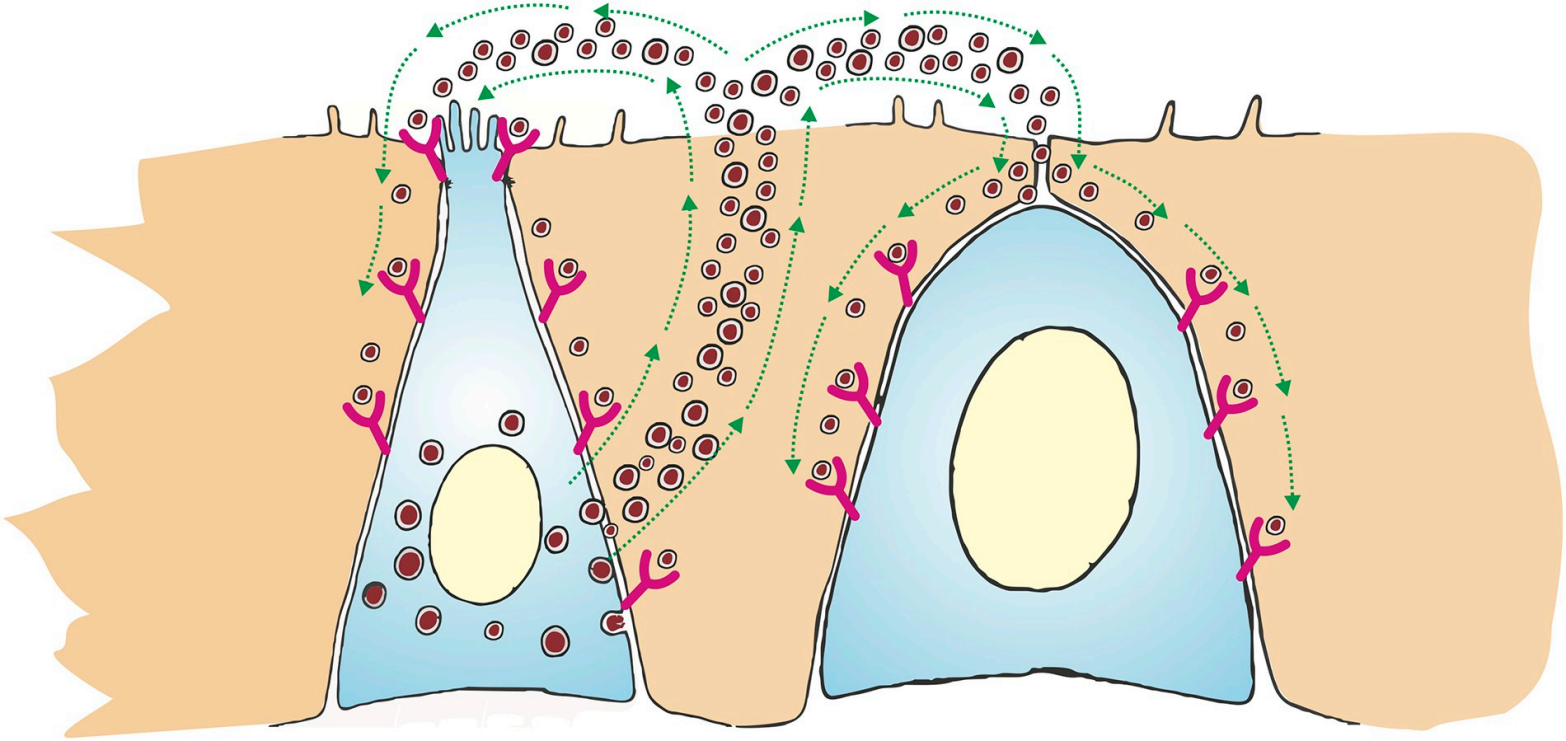
Schematic of paracrine and autocrine model. The secretory peptides (bombesin, glucagon, somatostatin, cholecystokinin and gastrin) or growth factors (platelet-derived growth factor, epidermal growth factor, transforming growth factor, insulin-like growth factor and fibroblast growth factor) produced by neuroendocrine cell tumors (Left cell) influence tissue growth with subsequent transformation into neoplastic cells (Right cell).

Other theories propose that the paracrine or autocrine growth loop effect by secretory peptides by neuroendocrine cell tumors (bombesin, glucagon, somatostatin, cholecystokinin and gastrin) or growth factors (platelet-derived growth factor, epidermal growth factor, transforming growth factor, insulin-like growth factor and fibroblast growth factor) produced by neuroendocrine cell tumors. These secretory peptides and growth factors are able to influence tissue growth with subsequent transformation into neoplastic cells [46,103,104] (Fig 2).

Several neuroendocrine cell secretory peptides have been described over the years [105]. The expression of these secretory peptides receptors has been reported in normal tissue and in diverse tumors as the SPM found in our study, such as GIT adenocarcinomas (esophagus, gastric, small intestine, colorectal and pancreatic), GIST, hepatocellular carcinoma, non-small cell lung cancer, thyroid cancer, and NENs [106–116]. Our findings may support the hypothesis of paracrine effect of secretory peptides by neuroendocrine cell tumors with the SPM. We found association between adenocarcinomas and NENs in the GIT (p:<0.0001) and adenocarcinomas and NENs in the same GIT segment (p<0.001).

We would like to acknowledge the limitations of our study. First, in some articles the final diagnosis of SPM was not made with immunohistochemistry. Second, in some articles patients’ follow up was not enough to determine the progression of disease. Finally, systematic reviews of case reports delineate a number of challenges to research on rare and heterogeneous conditions. However we consider them as important tools for initial data sources of such conditions and move from the anecdote to the evidence [117].

## Conclusion

Our systematic review provides a comprehensive review of SPM associated with NENs. With our data we cannot conclude the actual prevalence of NENs with SPM synchronic due to type of study, but we can conclude that the presence of SPM synchronic with NENs is not a rare condition. Large numbers of cases have documented the presence of NETs G1 (carcinoids) in GIT with synchronous SPM in a range between 62–88%. Prospective studies are necessary to determinate the relationship between paracrine effect by NENs and develop of SPM.

## Supporting information

S1 Table Characteristics of patients included in the study. (XLSX)

S2 Table Quality assessment of case reports. (PDF)

S3 Table Characteristics of patients with more than one SPM. (PDF)

S1 PRISMA Checklist PRISMA checklist. (DOC)

## Abbreviations

GIT: Gastrointestinal tract
NEC: Neuroendocrine carcinoma
NEN: Neuroendocrine neoplasms
NETs: Neuroendocrine tumors
PRISMA: Preferred Reporting Items for Systematic reviews and Meta-Analysis
SPM: Secondary primary malignancies
