# Supplementary material for: Neuroendocrine neoplasms of gastrointestinal tract and secondary primary synchronous tumors: A systematic review of case reports. Casualty or causality?
Source: PLoS One. 2019 May 14;14(5):e0216647. doi: 10.1371/journal.pone.0216647 (PMC6516644; doi:10.1371/journal.pone.0216647)
Supplement: S2 Table — (PDF) [file pone.0216647.s002.pdf]

S2 Table. Quality assessment of case reports

Localitation	Author	Year	Adequate description	Reliable outcome	Convincing evidence
Esophagous					
	Saw E.	1997	YES	YES	YES
	Deepak P.	2011	YES	YES	YES
	Fan H.	2017	YES	YES	Partially
Stomach					
	Rassidakis GZ	1998	YES	YES	YES
	Cunha P.	1998	YES	YES	NO
	Yang L.	2006	YES	YES	YES
	Sawalakhe NR.	2009	YES	YES	YES
	Moya Valverde E.	2009	YES	YES	NO
	Cirillo F.	2010	YES	YES	Partially
	Cirillo F.	2010	YES	YES	Partially
	Samaras VD.	2011	YES	YES	YES
	Duman DG.	2012	YES	YES	YES
	Nakayama Y.	2012	YES	YES	YES
	Ding J.	2014	YES	YES	YES
	Herreros-Villanueva M.	2016	YES	YES	YES
Small intestine (Duodenum, ampulla of vater, jejunum and ileum					
	Karatzas G.	2000	YES	YES	Partially
	McCabe H.	2001	YES	YES	YES
	Koçer NE.	2007	YES	YES	YES

	Srilatha PS.	2007	YES	YES	NO
	Sen N.	2008	YES	YES	YES
	Athanasopoulos PG.	2011	YES	YES	YES
	Martínez MM.	2011	YES	YES	YES
	Pusiol T.	2011	YES	YES	YES
	Cokmert S.	2013	YES	YES	YES
	Kaur R.	2013	YES	YES	YES
	Fukaya M	2014	YES	YES	NO
	Hsu A.	2015	YES	YES	YES
	Deng XF.	2015	YES	YES	YES
	Rivadeneira D.	1996	YES	NO	NO
	Tessier DJ.	2002	YES	Partially	No
	Buragas M.	2005	YES	YES	Partially
	Cioffi U.	2005	YES	YES	YES
	Reim D.	2008	YES	YES	YES
	Aslam M.	2009	YES	YES	YES
	McHugh S	2009	YES	YES	NO
	Boltin D.	2011	YES	YES	YES
	Wohadlo Ł.	2011	Partially	YES	No
	Berroa de la Rosa E.	2014	YES	YES	YES
	Grace S.	2015	YES	YES	YES
	Waldon K.	2015	YES	YES	YES
	Almajano EA.	2016	YES	YES	YES
	Gray S.	2016	YES	YES	YES
	Tsunenari T.	2016	YES	YES	YES
	Dafashy TJ.	2016	YES	YES	NO
	Kim SH.	2017	YES	YES	NO
	Shan B.	2017	YES	YES	YES

Large intestine (appendix, colon, rectum)					
	Zirkin H.	1996	YES	YES	YES
	Ganguly S.	2006	YES	YES	Partially
	Park JS.	2010	YES	YES	YES
	Salemis NS.	2010	YES	YES	YES
	Adams BN.	2011	YES	YES	Partially
	Lendzion R.	2015	YES	YES	YES
	Meeks MW.	2016	YES	YES	YES
	Nakayama Y.	2013	YES	YES	YES
	Lipka S.	2014	YES	YES	YES
	Xu F.	2014	YES	YES	Partially
	Zhu JG.	2015	YES	YES	YES
	Athiyappan K.	2015	YES	YES	YES
	Mohapatra M.	2016	YES	YES	YES
	Vootla V.	2016	YES	YES	YES
	Zukanović G.	2016	YES	YES	YES
	Winn JN.	2017	YES	YES	YES
	Winn JN.	2017	YES	Partially	NO
