# Supplementary material for: Neuroendocrine neoplasms of gastrointestinal tract and secondary primary synchronous tumors: A systematic review of case reports. Casualty or causality?
Source: PLoS One. 2019 May 14;14(5):e0216647. doi: 10.1371/journal.pone.0216647 (PMC6516644; doi:10.1371/journal.pone.0216647)
Supplement: S3 Table — (PDF) [file pone.0216647.s003.pdf]

S3 Table. Characteristics of patients with more than one SPM

Author	Year	Age	Gender	Localization neuroendocrine tumor	Family (Grade)	Localization SPM	Type of tumor	NETs Metastases	SPM N
Fan H.	2017	53	Female	Esophagus	NEC (Small cell)	Esophagus	Squoamous cell carcinoma	No	No
						Stomach	Adenocarcinoma		
						Stomach	GIST		
Herreros-Villanueva M.	2016	48	Male	Stomach	NEC (Large cell)	Stomach	Adenocarcinoma	No	Yes
						Stomach	MALT		
Welter HF.	1980	NA	NA	Small Intestine	NET (G1)	Colon	Unclassifiable carcinomas	NA	NA
						Pancreas	Unclassifiable carcinomas		
Ott RA.	1987	60	Male	Ileum	NET (G1)	Rectum	Adenocarcinoma	Yes	No
						Prostate	Adenocarcinoma		
						stomach	GIST		
						Lung	Angiosarcoma		
Martínez MM.	2011	52	Female	Duodenum	NET (G1)	Duodenum	Adenocarcinoma	No	No
						Stomach	GIST		
Fukaya M	2014	69	Male	Ampulla of vater	NEC (Small cell)	Stomach	Adenocarcinoma	No	Yes
						Esophagus	Squamous cell carcinoma		
Takasima S.	1983	71	Male	Rectum	NET (G1)	Esophagus	Nonclassifiable carcinoma	No	No
						Ureter	Unclassifiable carcinomas		
Kim SH.	2017	52	Male	Jejunum	NET (G1)	Jejunum	Adenocarcinoma	No	No
						Colon/Rectum	Adenocarcinoma		
						Colon/Rectum	Adenocarcinoma		
